# Association of Television Viewing Time with Body Composition and Calcified Subclinical Atherosclerosis in Singapore Chinese

**DOI:** 10.1371/journal.pone.0132161

**Published:** 2015-07-01

**Authors:** Ei Ei Khaing Nang, Rob M. van Dam, Chuen Seng Tan, Falk Mueller-Riemenschneider, Yi Ting Lim, Kai Zhi Ong, Siqing Ee, Jeannette Lee, E. Shyong Tai

**Affiliations:** 1 Saw Swee Hock School of Public Health, National University of Singapore, Singapore, Republic of Singapore; 2 Department of Medicine, Yong Loo Lin School of Medicine, National University of Singapore, Singapore, Republic of Singapore; 3 Department of Nutrition, Harvard School of Public Health, Boston, Massachusetts, United States of America; 4 Institute for Social Medicine, Epidemiology and Health Economics, Charite Univeristy Medical Centre, Berlin, Germany; 5 Yong Loo Lin School of Medicine, National University of Singapore, Singapore, Republic of Singapore; Graduate School of Medicine, Osaka University, JAPAN

## Abstract

**Objective:**

Sedentary behavior such as television viewing may be an independent risk factor for coronary heart disease. However, few studies have assessed the impact of television viewing time on coronary artery calcification and it remains unclear how body fat contributes to this relationship. The aim of this study is to evaluate the association between television viewing time and subclinical atherosclerosis and whether effects on visceral or subcutaneous fat may mediate any associations observed.

**Methods:**

This was a cross-sectional study of 398 Chinese participants (192 men and 206 women) from Singapore prospective study. Participants were free from known cardiovascular diseases and underwent interview, health screening, computed tomography scans of coronary arteries and abdomen. Spearman’s correlation was used to test the correlation between television viewing time, physical activity, body composition and abdominal fat distribution. The association between television viewing time and subclinical atherosclerosis was assessed by multiple logistic regression analysis.

**Results:**

In men, television viewing time was significantly correlated with higher body fat mass index, percent body fat, subcutaneous and visceral fat. These associations were in the same direction, but weaker and not statistically significant in women. Television viewing time (hours/day) was associated with subclinical atherosclerosis in men (odds ratio: 1.41, 95% CI: 1.03-1.93) but no significant association was observed in women (odds ratio: 0.88, 95% CI: 0.59-1.31) after adjusting for potential socio-demographic and lifestyle confounders. Further adjustments for biological factors did not affect these associations.

**Conclusions:**

Television viewing time was associated with greater adiposity and higher subcutaneous and visceral fat in men. TV viewing time was also associated with subclinical atherosclerosis in men and the potential mechanisms underlying this association require further investigation.

## Introduction

Previous studies had found that sedentary behavior is associated with incident cardiovascular diseases (CVDs) and cardiovascular mortality [1,2]. However, different sedentary behaviors may have different effects. In several studies, the association between health and television (TV) viewing time was stronger than for other types of sedentary behavior [3–8].

We previously reported that longer TV viewing time but not other forms of sedentary behavior were associated with higher body mass index (BMI) and insulin resistance [9]. Interestingly, in our study, the association with insulin resistance was only partly mediated by BMI [9]. However BMI may not fully capture the effects of sedentary behavior on body composition. Body weight comprises a combination of lean mass and fat mass. It is not clear whether TV viewing time affects one or both of these parameters. In addition, BMI does not capture fat distribution. Specifically, it does not differentiate between subcutaneous and visceral fat mass—the latter may be particularly relevant to insulin resistance and the development of CVDs [10–12].

Although several studies have shown an association between TV viewing time and CVD risk factors, only few studies have clearly shown an association with CVDs [7,13–15]. None of these have been conducted in Asian settings, which may be particularly pertinent as these populations undergo rapid socio-economic development and adopt sedentary lifestyles. In addition, there were conflicting results for the association between TV viewing time and subclinical atherosclerosis [16–20].

Thus, the aims of this study were to determine the association between TV viewing time and body composition, particularly lean mass versus fat mass and the distribution of body fat between the subcutaneous and visceral fat compartments and to determine the association between TV viewing time and subclinical atherosclerosis. We also sought to determine if these associations were independent of measures of physical activity and if these associations were mediated through body fatness and conventional cardiovascular risk factors.

## Materials and Methods

### Study design and study population

**Fig 1:**
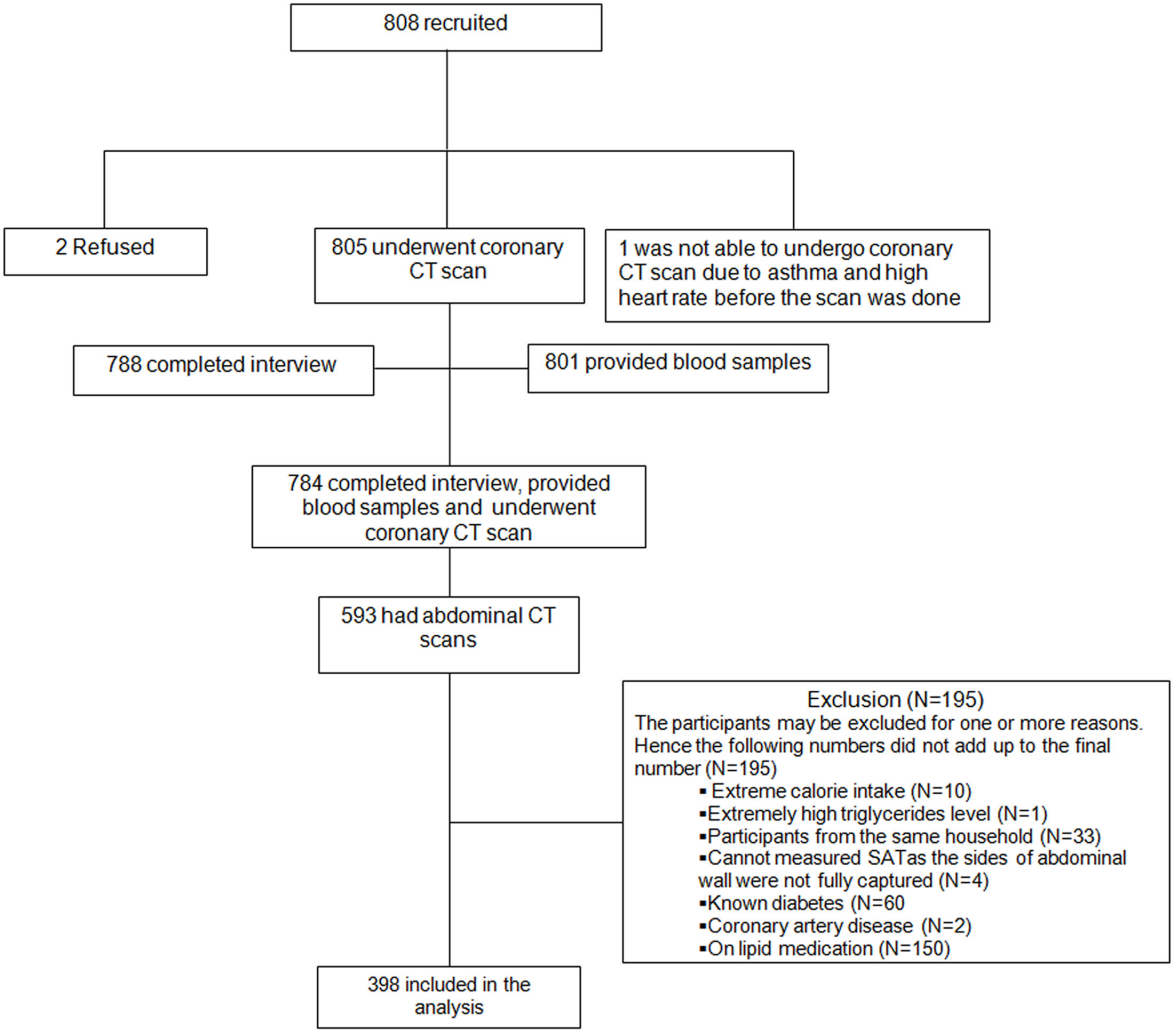
The flow chart of participants considered including in the analysis.

This analysis was based on cross sectional data of 398 participants who participated in a follow-up study of the Singapore prospective study [21]. From the participants of the follow-up study, the first 808 participants who met the eligibility criteria (aged above 50 years old, who did not have a history of heart failure, heart attack, stroke, kidney failure, cancer, and were not treated with high-dose steroids) were invited to participate in this study. The flowchart of participants included for the analysis is shown in Fig 1. Of the 808, 2 refused and 1 was unable to undergo the computed tomography (CT) scan due to asthma and a high heart rate before the scan was done. The participants who agreed to participate were then invited to undergo the interview and health examination including CT scan on different days. Of the 805 who underwent coronary CT scans, 788 completed the questionnaire and 801 provided blood samples. A total of 784 participants completed the questionnaire, provided blood samples and underwent coronary CT scans. Out of 784, 593 participants also had abdominal CT scans, all of whom were Chinese. The reason for the difference in this number is that the abdominal CT scans were carried out as part of a separate grant that was funded only after the initial study was initiated. As such, only a subset of the subjects had abdominal scans. From these 593, we excluded individuals who had extreme values for calorie intake which were above or below the 3 standard deviations (SD) from the mean intake by gender (N = 10), extremely high triglycerides level (>12.4 mmol/L) (N = 1), participants from the same household (N = 33) and participants with missing subcutaneous adipose tissue (SAT) measurements as the sides of abdominal wall were not fully captured (N = 4). We also excluded participants with known diabetes (N = 60), coronary artery disease (N = 2), and on lipid medication (N = 150) to avoid re-call bias resulting from differential recall in patients who have been advised to change their physical activity pattern for their medical conditions. Participants could be excluded for one or more reasons. A total of 398 participants were included in the analysis.

### Ethics statements

Ethics approval was obtained from 2 institutional review boards (the National University of Singapore and the Singapore General Hospital). Written informed consent was obtained from each participant before the study was conducted.

### Assessment of TV viewing time

TV viewing time was assessed by asking participants “Please estimate the total time during the last week that you spent watching TV or video” and it was recorded in hours, separately for weekdays and weekends and average hours of TV viewing time per day were calculated.

### Assessment of coronary artery calcium

Coronary artery calcium (CAC) was assessed by a 64-slicer multi-detector GE Light Speed volume CT (GE Medical Systems, Milwaukee, Wisconsin). The total CAC score was calculated according to the Agatston-Janowitz 130 score [22].

### Assessment of body composition and distribution of abdominal fat

Fat mass (FM), lean mass (LM) and total body fat percent (%BF) were assessed by dual-energy x-ray absorptiometry (DXA) imaging (Discovery Wi; Hologic, Bedford, MA and software Hologic Apex 3.01) using a medium speed total body acquisition mode. We used fat mass index (FMI) and lean mass index (LMI) as proposed by Vanltaiie et al because similar to BMI these indexes are independent of height and thus easier to interpret body composition data [23]. FMI and LMI were calculated by dividing FM (kg) and LM (kg) by the square of height (m^2^) respectively.

CT scans of the abdomen were carried out to measure the abdominal adipose tissues. A single observer identified inter-vertebral space L2/L3 and L4/L5 levels by efilm work station version 4.0 (Hartland, USA). Areas of subcutaneous adipose tissue (SAT) and visceral adipose tissue (VAT) at L2/L3 and L4/L5 levels were measured by sliceOmatic version 5.0 (Tomovision, Magog, Canada) using attenuation range of -190 to -30 Hounsfield units. VAT was defined as adipose tissue inside the abdominal muscular wall and SAT as adipose tissue outside the abdominal muscular wall [24]. Each image was analysed by two independent readers and the mean of the two readings was taken as final measurement. Average inter-observer coefficients of variation for SAT at L2/L3 and L4/L5 were 2.34% and 1.80% and for VAT at L2/L3 and L4/L5 were 3.22% and 3.13% respectively. In this study, we presented SAT and VAT at L2/L3 because VAT at L2/L3 was shown to be the best level to estimate the total VAT volume and better correlated with cardiovascular risk factors than VAT at L4/L5 in Asian Chinese [25].

### Assessment of risk factors

Two readings of blood pressure were taken from participants using digital blood pressure monitor (Dinamap Pro100V2) and mean value of the two readings was calculated. If the difference between two readings was greater than 10mmHg for systolic blood pressure (SBP) and/or greater than 5mmHg for diastolic blood pressure (DBP), a third reading was taken and mean value of the second and the third readings was calculated.

For blood tests, participants were informed to fast overnight for at least 10 hours. Venous blood was drawn and collected in plain, EDTA, and fluoride oxalate tubes. Blood tubes were left to stand for 30 minutes at room temperature before storing them at 4°C. The samples were sent to Singapore general hospital laboratory for analysis where serum was tested for creatinine, glucose, total cholesterol, high density lipoprotein cholesterol (HDL-C), low density lipoprotein cholesterol (LDL-C) and triglycerides (TG) using a chemistry analyzer (Beckman Coulter Unicel DxC 800). Glycated haemoglobin A1C (HbA1c) levels were measured using immunoassay (Roche Cobas c501).

We calculated the ratio of triglyceride to HDL-C (TG/HDL-C), which we considered as a risk factor for coronary artery disease as well as a surrogate marker for insulin resistance [26,27].

### Assessment of covariates

Demographic, physical activity, dietary intake, medical history and other lifestyle factors were assessed by an interviewer-administered questionnaire.

Dietary intake was assessed by a semi-quantitative 169-item validated food frequency questionnaire that was also used in the National Nutrition Surveys [28]. The percentages of energy intake derived from carbohydrate and protein were calculated as the calories from the macronutrient divided by total calorie intake.

Physical activity was assessed by a validated physical activity questionnaire which covered transportation, leisure time, occupation and household activities [29]. The questionnaire had been validated with accelerometer in Singapore population and had reasonable validity and reliability [29]. The detailed method of assessing physical activity was described elsewhere [29,30]. Briefly, the participants reported the type, frequency and duration of various activities in the transportation, occupation, leisure time, and household domain. Transportation activities included walking and cycling; occupational activity included light, moderate and vigorous occupational activity; leisure time activities included 48 specific activities; household activities included 15 specific activities; and for leisure and household activities the questionnaire also included open-ended questions. A metabolic equivalent of task (MET) value was assigned to each type of reported activity according to the 2011 compendium of physical activity [9,31]. Physical activity level per week for each activity was calculated as frequency per week x duration in hours per day x intensity (METs) allowing physical activity to be quantified as MET-hour/week.

### Definitions

Hypertension was defined as a systolic blood pressure greater than 140 mm Hg or a diastolic blood pressure greater than 90 mm Hg or a history of hypertension or current use of antihypertensive medications.

For subclinical atherosclerosis, we used the cutoff value of 100 and above for CAC score which has been shown to be associated with intermediate to high risk for coronary heart disease events [32–34] and it was also used in other studies to define subclinical atherosclerosis [35–37].

Activities between 1.6 to 2.9 METs were classified as light-intensity; those between 3 to 5.9 METs were classified as moderate-intensity and those ≥6 METs were classified as vigorous-intensity [31].

### Statistical analysis

Previous studies have shown that the association between TV viewing time and CVD risk factors was different between men and women [38–40]. Hence, we first tested for effect modification of the association by gender by including the interaction term gender x TV viewing time in the models and the significance was assessed by Wald test. There was significant interaction between TV viewing time and gender for subclinical atherosclerosis (p value = 0.03). Hence, we stratified all the analysis by gender.

For Tables 1 and 2, we calculated proportions for categorical variables by the status of subclinical atherosclerosis in men (Table 1) and women (Table 2). Pearson’s chi-square test and Fisher’s exact test were used to compare proportions between the groups where appropriate. For continuous variables which were normally distributed, we summarized them with the means and standard deviations, and student’s t-test was used to compare the difference between the groups. For those which were not normally distributed, we calculated the medians and inter-quartile ranges, and Wilcoxon rank-sum test was used to assess the difference between the groups. For Table 3, spearman’s rank partial correlations between TV viewing time/physical activities with measures of body composition were calculated. Average TV viewing time was adjusted for age, and light to vigorous physical activities. Light to vigorous physical activities were adjusted for age, average TV viewing time, and other corresponding levels of intensity of physical activities except total level of physical activity. Total physical activity was adjusted for age and average TV viewing time. For Table 4, multiple logistic regression analysis was used to assess the association between TV viewing time and subclinical atherosclerosis and the results were presented in odds ratio (OR) with the 95% confidence interval (CI). The crude association between TV viewing time and subclinical atherosclerosis was assessed with model 1. Model 2 adjusted the association for light, moderate and vigorous physical activities. Model 3 adjusted the association for variables in model 2 plus age, cigarette smoking (never smoker and ever smoker), current alcohol drinking (yes/no), occupational sitting time (hours/day) and employment status (yes /no), and model 4 adjusted the association for the variables in model 3 plus dietary intake (total calorie intake, polyunsaturated/saturated fat, protein, carbohydrate and fibre intake). Model 5 further adjusted for VAT and SAT to examine if these were the mediators for the observed associations. Additional adjustment for hypertension (yes /no), HbA1c, TG/HDL-C, and LDL-C was performed in model 6 to assess the potential mediation effect of these biomarkers. P-values <0.05 were considered statistically significant in all analysis.

**Table 1:** Characteristics of the study population by subclinical atherosclerosis status in men.

	Subclinical atherosclerosis		
	No	Yes	p value
**N (%)**	152 (79.17)	40 (20.83)	
Age, years (mean ±SD)	57.16 ± 5.52	60.95 ± 7.33	**<0.01**
**Highest level of education (N, %)**			
None/ primary	33 (21.71)	12 (30.00)	0.73
Secondary	60 (39.47)	15 (37.50)	
Technical school	32 (21.05)	7 (17.50)	
University	27 (17.76)	6 (15.00)	
Employed (N, %)	128 (84.21)	30 (75.00)	0.18
**Smoking (N, %)**			
Never smoker	80 (52.63)	20 (50.00)	0.77
Ever smoker	72 (47.37)	20 (50.00)	
Hypertension (history or newly diagnosed) (N, %)	74 (48.68)	22 (55.00)	0.48
Current alcohol drinking (N, %)	44 (28.95)	11 (27.50)	0.86
Average TV viewing time (hours/day) (mean ±SD)	2.01 ±1.22	2.53 ±1.67	**0.03**
Average occupational sitting time (hours/day)(median, IQR)	0 (0, 2.63)	0 (0, 2.32)	0.30
Light physical activity level (MET-hour/week) (median, IQR)	49.20 (13.87, 84.26)	34.33 (19.09,72.46)	0.41
Moderate physical activity level (MET-hour/week) (median, IQR)	15.75 (5.34, 47.75)	28.82 (10.33, 60.33)	0.13
Vigorous physical activity level (MET-hour/week) (median, IQR)	0 (0, 9.00)	0 (0, 0)	0.10
Total physical activity level (MET-hour/week) (median, IQR)	104.39 (57.15, 139.07)	94.98 (64.17, 141.83)	0.95
HbA1c (%) (mean ±SD)	5.63 ± 0.49	6.15 ± 1.28	**<0.01**
TG/HDL (median, IQR)	0.86 (0.57, 1.37)	1.45(0.83, 2.07)	**<0.01**
LDL-C measured (mmol/L) (mean ±SD)	3.67 ± 0.85	3.88 ± 0.77	0.15
Fat mass index (kg/m^2^) (mean ±SD)	6.69 ± 1.79	7.11 ± 2.06	0.20
Lean mass index (kg/m^2^) (mean ±SD)	16.95 ± 1.84	17.25 ± 1.84	0.36
Total Body % Fat (mean ±SD)	27.9 ± 4.71	28.73 ± 5.09	0.33
SAT at L2/L3 (cm^2^) (mean ±SD)	91.36 ± 42.46	93.90 ± 42.05	0.74
VAT at L2/L3 (cm^2^) (mean ±SD)	130.44 ± 66.42	156.73 ± 71.27	**0.03**

IQR = inter-quartile range; TG = triglycerides; HDL-C = High density lipoprotein cholesterol; LDL-C = Low density lipoprotein cholesterol; HbA1c = glycated haemoglobin A1C; SAT = subcutaneous adipose tissue; VAT = visceral adipose tissue.

**Table 2:** Characteristics of the study population by subclinical atherosclerosis status in women.

	Subclinical atherosclerosis		
	No	Yes	p value
**N (%)**	187 (90.78)	19 (9.22)	
Age, years (mean ±SD)	56.58 ± 5.57	60.21 ± 7.08	**<0.01**
**Highest level of education (N, %)**			
None/ primary	49 (26.20)	6 (31.58)	0.56¥
Secondary	95 (50.80)	11 (57.89)	
Technical school	33 (17.65)	1 (5.26)	
University	10 (5.35)	1 (5.26)	
Employed (N, %)	100 (53.48)	12 (63.16)	0.42
**Smoking (N, %)**			
Never smoker	173 (92.51)	18 (94.74)	1.00¥
Ever smoker	14 (7.49)	1 (5.26)	
Hypertension (history or newly diagnosed) (N, %)	79 (42.25)	13 (68.42)	**0.03**
Current alcohol drinking (N, %)	16 (8.56)	0 (0)	0.37¥
Average TV viewing time (hours/day) (mean ±SD)	2.50 ±1.65	2.12 ±1.38	0.33
Average occupational sitting time (hours/day) (median, IQR)	0 (0, 0.36)	0 (0, 0.39)	0.91
Light physical activity level (MET-hour/week) (median, IQR)	57.43 (34.75, 90.33)	59.25 (32.98, 116.98)	0.42
Moderate physical activity level (MET-hour/week) (median, IQR)	19.25 (7.00, 36.75)	14.00 (1.75, 30.50)	0.33
Vigorous physical activity level (MET-hour/week) (median, IQR)	0 (0, 0.63)	0 (0, 0)	0.42
Total physical activity level (MET-hour/week) (median, IQR)	95.00 (60.37, 132.7)	99.52 (59.25, 133.10)	0.84
HBA1c (%) (mean ±SD)	5.69 ± 0.55	6.02 ± 0.93	**0.02**
TG/HDL (median, IQR)	0.62 (0.40, 0.94)	0.82 (0.50, 1.34)	0.26
LDL-C measured (mmol/L) (mean ±SD)	3.84 ± 0.84	3.83 ± 1.05	0.93
FMI (kg/m^2^) (mean ±SD)	9.02 ± 2.13	9.90 ± 2.54	0.09
LMI (kg/m^2^) (mean ±SD)	13.74 ± 1.39	14.39 ± 1.55	0.06
Total Body % Fat (mean ±SD)	39.18 ± 4.65	40.23 ± 4.40	0.35
SAT at L2/L3 (cm^2^) (mean ±SD)	129.22 ± 48.73	136.92 ± 41.51	0.51
VAT at L2/L3 (cm^2^) (mean ±SD)	80.14 ± 46.50	97.60 ± 51.56	0.12

IQR = inter-quartile range; TG = triglycerides; HDL-C = High density lipoprotein cholesterol; LDL-C = Low density lipoprotein cholesterol; HbA1c = glycated haemoglobin A1C; FMI = Fat mass index, LMI = Lean mass index, SAT = subcutaneous adipose tissue; VAT = visceral adipose tissue.

^¥^ Fisher exact test.

**Table 3:** Partial correlation of TV viewing time and physical activity with body composition.

	FMI (kg/m^2^)	LMI (kg/m^2^)	Total Body % Fat	SAT at L2/L3 (cm^2^)	VAT at L2/L3 (cm^2^)
**Men (N = 192)**					
Average TV viewing time (hours/day)	**0.15 (0.04)**	0.02 (0.75)	**0.16 (0.02)**	**0.15 (0.03)**	**0.15 (0.04)**
Light physical activity level (10MET-hour/week)	0.02 (0.83)	0.11 (0.12)	-0.01 (0.93)	0.004 (0.96)	0.08 (0.27)
Moderate physical activity level (10MET-hour/week)	-0.002 (0.98)	**0.24 (0.001)**	-0.07 (0.31)	0.01 (0.87)	0.07 (0.35)
Vigorous physical activity level (10MET-hour/week)	0.03 (0.66)	**0.21 (0.003)**	-0.07 (0.35)	-0.03 (0.70)	-0.01 (0.90)
Total physical activity level (10MET-hour/week)	-0.08 (0.28)	**0.22 (0.002)**	**-0.19 (0.008)**	-0.10 (0.18)	-0.02 (0.74)
**Women (N = 206)**					
Average TV viewing time (hours/day)	0.11 (0.14)	0.10 (0.16)	0.12 (0.10)	0.07 (0.35)	0.12 (0.10)
Light physical activity level (10MET-hour/week)	0.02 (0.73)	0.13 (0.07)	-0.02 (0.73)	0.06 (0.44)	0.09 (0.23)
Moderate physical activity level (10MET-hour/week)	-0.04 (0.54)	0.06 (0.42)	-0.11 (0.11)	-0.09 (0.22)	-0.01 (0.91)
Vigorous physical activity level (10MET-hour/week)	-0.09 (0.19)	0.05 (0.50)	**-0.16 (0.02)**	-0.12 (0.09)	-0.09 (0.22)
Total physical activity level (10MET-hour/week)	0.04 (0.59)	**0.16 (0.02)**	-0.06 (0.36)	0.06 (0.39)	0.08(0.24)

Partial spearman’s rank correlation reported and p value in the bracket.

Average TV viewing time was adjusted for age, and light to vigorous physical activities.

Light to Vigorous physical activities were adjusted for age, average TV viewing time, and other corresponding levels of physical activities.

Total Physical activity was adjusted for age and average TV viewing time.

FMI = Fat mass index, LMI = Lean mass index, %BF = % Body fat, SAT = subcutaneous adipose tissue.

VAT = visceral adipose tissue, L2/L3 = inter-vertebral space at L2/L3.

**Table 4:** Association of TV viewing time (hours/day) and subclinical atherosclerosis.

	Subclinical atherosclerosis					
	In Men			In Women		
	OR	95% CI		OR	95% CI	
Average TV viewing time (per hours/day)						
Model 1	**1.31** *	**1.02**	**1.68**	0.85	0.61	1.18
Model 2	**1.32** *	**1.02**	**1.70**	0.85	0.60	1.20
Model 3	**1.35** *	**1.01**	**1.81**	0.90	0.61	1.31
Model 4	**1.41** *	**1.03**	**1.93**	0.88	0.59	1.31
Model 5	**1.38** *	**1.00**	**1.90**	0.83	0.54	1.30
Model 6	**1.45** *	**1.04**	**2.02**	0.85	0.52	1.37

Model 1: Unadjusted.

Model 2: Adjusted for light, moderate and vigorous physical activities.

Model 3: Model 2 plus additional adjustment for age, cigarette smoking, current alcohol drinking, occupational sitting time and employment status.

Model 4: Model 3 plus additional adjustment for total calorie intake, polyunsaturated fat: saturated fat, protein, carbohydrate and fibre intake.

Model 5: Model 4 plus additional adjustment for visceral adipose tissue and subcutaneous adipose tissue.

Model 6: Model 5 plus additional adjustment for hypertension, HbA1c, TG/HDL-C, and LDL-C.

*p value<0.05.

## Results

Tables 1 and 2 show the characteristics of participants according to subclinical atherosclerosis status in men and women respectively. The proportions of men and women in our samples are quite similar; 192 men (48.2%) and 206 women (51.8%). However, there was fewer percentage of subclinical atherosclerosis in women than men (9.2% vs 20.8%). Those who had subclinical atherosclerosis were older and had higher level of HbA1c in both men and women. They also had longer hours of TV viewing time and higher levels of TG/HDL-C and VAT in men (Table 1) and were more likely to have hypertension in women (Table 2).

The partial correlations of physical activity and TV viewing time with body composition and abdominal fat are presented in Table 3. In men, TV viewing time was significantly correlated with FMI, %BF, SAT and VAT. Moderate, vigorous and total physical activity was positively correlated with LMI. In addition, total physical activity was negatively correlated with %BF. In women, only vigorous physical activity was negatively correlated with %BF and total physical activity was positively associated with LMI. The correlations of TV viewing time with body composition and abdominal fat were not significant in women. TV viewing time was also not correlated with LMI in both men and women (Table 3). Correlations of TV viewing time and physical activity with SAT and VAT at L4/L5 were similar as that of L2/L3 (data not shown).

Table 4 shows the association of TV viewing time with subclinical atherosclerosis. Higher TV viewing time was significantly associated with risk of subclinical atherosclerosis in men (OR: 1.31, 95%CI: 1.02–1.68) but not in women (OR: 0.85, 95%CI: 0.61–1.18) with no adjustment for confounders. Adjusting for physical activities in model 2, socio-demographic and lifestyle factors in model 3 and model 4 did not affect the observed association. Additional adjustment for VAT and SAT in model 5 also did not alter the association in both men (OR: 1.38, 95% CI: 1.00–1.90) and women (OR: 0.83, 95% CI: 0.54–1.30). Further adjustment for hypertension and biomarkers in model 6 also failed to affect the observed association and TV viewing time was independently associated with subclinical atherosclerosis in men (OR: 1.45, 95% CI: 1.04–2.02) and not in women (OR: 0.85, 95% CI: 0.52–1.37) after adjusting for all potential confounders (Table 4).

In addition to TV viewing time, age (OR: 1.12, 95% CI: 1.04–1.21) and HbA1c (OR: 2.91, 95% CI: 1.28–6.66) were independent predictors for subclinical atherosclerosis in men but age (OR: 1.16, 95% CI: 1.04–1.31) was the only independent predictor in women. The association of HbA1c and subclinical atherosclerosis was borderline significant (OR: 2.17, 95% CI: 0.98–4.85, p value = 0.06) in women (data not shown).

## Discussion

In our study, we found that TV viewing time was associated with adiposity in men which is consistent with our previous observation that TV viewing time was associated with BMI [9]. We have shown that the association between longer TV viewing time and higher BMI was largely due to an increase in fat mass. Furthermore, we have shown that this increase in adiposity affected both subcutaneous fat and visceral fat depots equally. Contrary to our initial hypothesis, TV viewing time did not show any association with reduced lean mass.

In addition, we showed that TV viewing time was independently associated with subclinical atherosclerosis in men after adjusting for other CVD risk factors. The finding in men was consistent with the finding from the Multi-Ethnic Study of Atherosclerosis study, in which sedentary behavior was associated with CAC among participants free of diagnosed cardiovascular diseases [17]. Similar finding was also observed in a study conducted in Japanese children, which found that TV viewing time was associated with carotid intima media thickness (IMT) which is also a marker for coronary atherosclerosis [20].

We found that longer TV viewing time was associated with increased VAT. In turn, VAT was associated with elevated CAC in men, in our study as well as in others studies [10,41,42]. However, this association between TV viewing time and VAT did not seem to explain the association between TV viewing time and subclinical atherosclerosis given that the latter association did not change much when adjusting for VAT. This association between TV viewing time and CAC that was independent of obesity was also seen in the Multi-Ethnic Study of Atherosclerosis study, in which the association between sedentary time and CAC persisted even after adjusting for BMI [17]. However, BMI is a rough estimate of body fatness and in this study we extend their findings by showing independence of directly measured body fat depots. Unfortunately, our study does not provide any mechanism by which TV viewing time may increase the risk of subclinical atherosclerosis and this would be an important subject of future studies.

In our study, we did not find any association of TV viewing time with subclinical atherosclerosis in women although the result from previous study found that TV viewing time was associated with incident CVD in an analysis without stratifying by gender [14]. However in our study, the number of women with subclinical atherosclerosis was much smaller than the number in men and the study may have lacked the statistical power to detect an association in women. Alternatively, it may be that CAC is not as effective for detecting subclinical atherosclerosis in women as in men. Previous data has shown that women, more than men, are likely to exhibit calcification in portions of the aorta that are not covered as part of the standard CAC [43]. This misclassification in women would result in weakening the association between TV viewing time and subclinical atherosclerosis in women.

In our study, we did not find significant associations between physical activity (irrespective of the level of intensity) and both SAT and VAT. Previously studies had shown that physical activity was inversely correlated with VAT and SAT [44,45]. This could be related to the fact that our participants had little engagement in vigorous physical activity. In line with this hypothesis, Sasai et al previously reported that vigorous activity but not light and moderate physical activity was associated with VAT and SAT [44]. In addition, our conflicting finding may be due to ethnic difference as Lesser et al reported that there was significant correlation between physical activity and SAT in Europeans but not in Chinese and South Asians [46].

We also did not find any association of physical activity with subclinical atherosclerosis in either men or women even though vigorous and total physical activities were correlated with higher LMI and lower %BF. Our study had limited power to detect this association as the number of persons with subclinical atherosclerosis was small, especially in women. Nonetheless, similar observations were made in the Prospective Army Coronary Calcium (PACC) study done in U.S which showed that physical activity was associated with favorable cardiovascular profile but not associated with calcified coronary atherosclerosis [47]. The authors suggested that the benefit of physical activity for reduction of CHD may be mediated through the stabilization of non-calcified plaques rather than calcified plaques [47]. In addition, in the Multi-Ethnic Study of Atherosclerosis, physical activity was not associated with subclinical vascular disease in a cross-sectional analysis [48,49]. Another possible alternative explanation for the lack of association of physical activity and CAC is the low participation in vigorous activity among study participants. Some previous studies which assessed the association between physical activity and subclinical atherosclerosis reported that only vigorous activity was associated with a lower risk [19,50]. Our participants were aged 50 years and older and 70% of study population did not participate in any vigorous intensity activity. Even though the participants were engaged in light and moderate intensity activities, the beneficial effects of light and moderate intensity activities on CVDs have been less consistent [51,52].

Our study has several strengths. To our knowledge, this is the first study that specifically examined the association between TV viewing time and calcified subclinical atherosclerosis and the role of visceral fat in this association. Previously studies have only examined sedentary behavior as a whole. We have previously shown that TV viewing may be different from other types of sedentary behavior. We were also able to take account of a wide range of potential confounders and mediators including diet, physical activity, fat distribution and cardio-metabolic biomarkers.

However, our study also has some limitations. Firstly, our study has modest power due to a limited number of cases. Secondly, TV viewing time and physical activity were self-reported. Hence there may be recall bias, measurement errors and misclassification since patients with CVDs might have been advised to exercise more, which may affect their recall of physical activity. To minimize this, we excluded individuals with known CVDs or diabetes, and those on lipid lowering medication. Since all individuals were asymptomatic, we believe that error in measurement of TV viewing time and physical activity would be non-differential and would therefore bias the association towards the null hypothesis of no association. As such, we feel that this bias is not likely to alter the conclusions of our study. Nonetheless, we accept that studies in which sedentary activity is more objectively measured would increase the certainty of these conclusions. Finally, although dietary intake was accounted in our study, we could not control sleep duration or quality. TV viewing time has been shown to be associated with short sleep, which was also independent risk factor for cardiovascular diseases [53].

## Conclusions

The findings from this study support that TV viewing time has detrimental effects on cardiovascular health independent of physical activity. In relation to obesity, we have preliminary finding that TV viewing time is associated with increased adiposity in both the subcutaneous and visceral fat compartments, but not with changes in lean mass. Even though VAT is associated with both TV viewing time and subclinical atherosclerosis, it may not be a substantial mediator. Thus, the association between TV viewing and coronary artery calcification remained unexplained and further research is needed to understand the pathway between TV viewing time and coronary artery calcification.
